# *ZmCOP1* Regulates Maize Mesocotyl Length and Plant Height through the Phytohormone Pathways

**DOI:** 10.3390/life13071522

**Published:** 2023-07-07

**Authors:** Liping Chen, Qiuhua Li, Ming Wang, Feng Xiao, Kangshi Li, Ran Yang, Meng Sun, Haiyan Zhang, Jinjie Guo, Jingtang Chen, Fuchao Jiao

**Affiliations:** 1 College of Agronomy, Qingdao Agricultural University, Qingdao 266109, China; 2 The Characteristic Laboratory of Crop Germplasm Innovation and Application, Provincial Department of Education, College of Agronomy, Qingdao Agricultural University, Qingdao 266109, China; 3 State Key Laboratory of North China Crop Improvement and Regulation, Hebei Sub-Center for National Maize Improvement Center, College of Agronomy, Hebei Agricultural University, Baoding 071001, China

**Keywords:** *ZmCOP1*, mesocotyl elongation, plant height, RNA sequencing, phytohormone

## Abstract

The morphogenesis of crops is critical to their yield performance. COP1 (constitutively photomorphogenic1) is one of the core regulators in plant morphogenesis and has been deeply studied in *Arabidopsis thaliana*. However, the function of COP1 in maize is still unclear. Here, we found that the mesocotyl lengths of *zmcop1* loss-of-function mutants were shorter than those of wild-type B73 in darkness, while the mesocotyl lengths of lines with *ZmCOP1* overexpression were longer than those of wild-type B104. The plant height with *zmcop1* was shorter than that of B73 in both short- and long-day photoperiods. Using transcriptome RNA sequencing technology, we identified 33 DEGs (differentially expressed genes) between B73′s etiolated seedlings and those featuring *zmcop1*, both in darkness. The DEGs were mainly enriched in the plant phytohormone pathways. Our results provide direct evidence that *ZmCOP1* functions in the elongation of etiolated seedlings in darkness and affects plant height in light. Our data can be applied in the improvement of maize plant architecture.

## 1. Introduction

Plants exhibit different morphological characteristics when grown in darkness and in light; together, these phenomenon is called morphogenesis [1,2]. The elongation of the hypocotyl or mesocotyl is critical to seedling emergence [3,4,5]. In *Arabidopsis thaliana*, COP1 (constitutively photomorphogenic 1) plays a central role in morphogenesis [6,7,8]. In darkness, the *Arabidopsis thaliana* hypocotyl elongates, while weak *cop1* mutants have shown a short hypocotyl length and open cotyledons [9].

In darkness, COP1 usually binds SPA1 (suppressor of phya 1) to form the COP1–SPA complex [10]. PIF1 (phytochrome interacting factor 1) interacts with the COP1–SPA complex to induce degradation of HY5 (elongated hypocotyl 5), resulting in hypocotyl elongation [11,12,13,14]. COP1 also participates in the degradation of WDL3 (wave-dampened 2-like 3) through the 26S proteasome-mediated pathway, leading to hypocotyl elongation [1,15].

In light, CRY (cryptochrome) inhibits the regulation activity of the COP1–SPA complex and stabilizes HY5 [16,17,18,19,20]. The Pfr (far-red light-absorbing form) of the photosensitive pigment induces phosphorylation of PIFs. Phosphorylated PIFs are recognized by COP1–SPA, then ubiquitinated and degraded by the 26S proteasome [14,21]. Thus, the stability of HY5 and the degradation of PIFs inhibit hypocotyl elongation. COP1 is also involved in the regulation of the circadian clock (Bhatnagar et al., 2020). A weak *cop1* mutant showed a short circadian clock gene expression cycle and an early flowering phenotype under short daylight [9,15,22,23,24].

Hormones such as BR (brassinolide), JA (jasmonic acid) and ETH (ethylene) play important roles in the regulation of morphogenesis. In *Arabidopsis*, BR promotes skotomorphogenesis. BRI1 (BR-insensitive1) and BIN2 (BR-insensitive2) promote accumulation of BBX28 and BBX29. BBX28 and BBX29 interact with BEE1 (BR-enhanced expression1), BEE2 (BR-enhanced expression2) and BEE3 (BR-enhanced expression3) [25]. HY5 enhances the activity of GSK3-like kinase BIN2 (brassinosteroid-insensitive 2) to repress skotomorphogenesis [24]. Ethylene inhibits EBF1 (ethylene response factor1) and EBF2 (ethylene response factor2) to stabilize EIN3 (ethylene-insensitive 3) and EIL1 (EIN3-like 1), respectively, inhibiting the opening and expansion of Arabidopsis cotyledons and maintaining skotomorphogenesis [26,27].

COP1 homologs have been identified in various plants, including *Arabidopsis thaliana*, *Sorghum bicolor*, *Zea mays* and *Oryza sativa* (Huai et al., 2020). Although *ZmCOP1* has been shown to restore the *atcop1-4* phenotypes in Arabidopsis, the function of *ZmCOP1* in maize has not been well studied. Here, we have phenotypically characterized *zmcop1* mutants and overexpression lines and identified DEGs between *zmcop1* mutants and wild types using RNA sequencing technology. We have shown that *ZmCOP1* may inhibit the elongation of the mesocotyl through the *BR* signal transduction pathway. We have provided evidence that *ZmCOP1* has a conserved function in *Zea mays* and *Arabidopsis thaliana*.

## 2. Materials and Methods

### 2.1. Plant Material and Growth Conditions

*zmcop1-1* and *zmcop1-2* mutants were collected from the maize EMS mutant library (www.elabcaas.cn, accessed on 14 September 2021). The wild type (B73 inbred line) from the same library was used as a control.

For the wild type (WT), *zmcop1-1* and *zmcop1-2*, a mesocotyl elongation analysis and an RT-qPCR analysis were performed. In a chi-square test, the WT and *zmcop1-1* were crossed to generate F_1_ hybrid lines; then, F_1_ hybrid seeds were self-fertilized to generate F_2_ lines. Around 140 seeds from one F_2_ ear were planted in soil in a greenhouse. The F_2_ seedlings were grown in the dark for 5–7 days. For plant height analysis, WT and *zmcop1-1* were planted in Jiaozhou (36.26429, 120.03192) in the summer of 2021, in Ledong (36.26429, 120.03192) in the winter of 2021 and in Jiaozhou again in the summer of 2022. The mesocotyl length and the plant height of the 7 day old etiolated seedlings were measured according to the standard methods.

*ZmCOP1* overexpression transgenic lines were generated by Beijing Bomei Xing’ao Technology Co., Ltd. In general, the *ZmCOP1* coding sequence was amplified and inserted into the 521 plasmid, furthered by the *ZmUBI* promoter. The transformation was performed following the standard Agrobacteria-mediated transformation protocol for maize, using B104 immature embryos. Positive transformation events were selected based on kanamycin and bar herbicide resistance. Positive transgenic lines were confirmed with PCR.

### 2.2. DNA Extraction and Genotyping

The CTAB method was used to extract the total DNA from leaves grown for 7 days. Two pairs of primers were designed near two mutation sites using primer5.0. To perform PCR, 2x Taq PCR StarMix with Loading Dye (GenStar) was used. A 1% agarose gel electrophoresis experiment was used for the last step of genotype detection.

### 2.3. Measurement of SPAD Value

The SPAD values (relative content of chlorophyll) of the ear leaf and the first leaf between the WT and *zmcop1* were measured with SPAD502 (Zhejiang Top Cloud-Agri Technology Co., Ltd., Hangzhou, China). The measuring position of the ear leaf was about 6 cm from the base of the first ear, and the measuring site of the first leaf was about 10 cm from the tip of the leaf. At least 15 groups of data were collected.

### 2.4. RNA Sequencing

#### 2.4.1. RNA Extraction

The construction of a cDNA library and an RNA sequencing analysis was completed with Wuhan MetWare Biotechnology Co., Ltd. (Wuhan, China).

Seven-day-old etiolated seedlings were collected for RNA isolation. Four biological replicates were used. The RNA purity was checked using a NanoPhotometer^®^ spectrophotometer (IMPLEN, Westlake Village, CA, USA). The RNA concentration was measured using a Qubit^®^2.0 Fluorometer (Thermo Fisher Scientific, Carlsbad, CA, USA). The RNA integrity was assessed using an RNA Nano 6000 Assay Kit of a Bioanalyzer 2100 system (Agilent Technologies, Santa Clara, CA, USA).

#### 2.4.2. Library Preparation for Transcriptome Sequencing

A total of 1 μg of RNA was used for each sample library preparation. Sequencing libraries were generated using a NEBNext^®^ Ultra^TM^ RNA Library Prep Kit for Illumina^®^ (New England Biolabs, Ipswich, MA, USA), following the manufacturer’s recommendations. Briefly, mRNA was purified from the total RNA using poly-T oligo attached magnetic beads. Fragmentation was carried out using divalent cations and under elevated temperatures in NEBNext First Strand Synthesis Reaction Buffer (5X). The first-strand cDNA was synthesized using a random hexamer primer and M-MuLV Reverse Transcriptase (RNase H-). Second-strand cDNA synthesis was performed using DNA Polymerase I and RNase H. cDNA fragments (preferentially 250~300 bp in length) were purified with an AMPure XP system (Beckman Coulter, Indianapolis, IN, USA). The PCR products were purified (AMPure XP system), and the library quality was assessed with the Agilent Bioanalyzer 2100 system.

#### 2.4.3. Clustering and Sequencing

Clustering of the index-coded samples was performed with a cBot Cluster Generation System using a TruSeq PE Cluster Kit v3-cBot-HS (Illumia, San Diego, CA, USA), according to the manufacturer’s instructions. After cluster generation, the library preparations were sequenced on an Illumina platform and 150 bp paired-end reads were generated.

#### 2.4.4. Analysis of the RNA-Seq Data

The raw sequencing data were filtered using fastp v0.19.3 with adapters and then aligned to Zm-B73-REFERENCE-NAM-5.0. The aligned reads were calculated with FeatureCounts v1.6.2. HISAT v2.1.0 was used to construct the index and to compare the clean reads to the reference genome [28].

Afterward, StringTie v1.3.4d was used for the prediction of new genes [29]. featureCounts v1.6.2 and StringTie v1.3.4d were used to calculate the gene alignment and the FPKM. DESeq2 v1.22.1 and edgeR v3.24.3 were used to analyze the differentially expressed genes between the two groups [30,31,32], and the *p*-values were corrected using the Benjamini–Hochberg method. The corrected *p*-values and |log2foldchange| were used as the thresholds for significant difference expression. A hypergeometric distribution test was used for KEGG and GO term analyses [33,34,35].

### 2.5. RT-qPCR Analysis

A SteadyPure plant RNA extraction kit (AG) was used to extract the total RNA. MonScript^TM^ RTIII ALL-in-One Mix with dsDNase (Monad) was used for reverse transcription. SYBR Green Pro Taq (AG) was used with an ABI 7500 Real-Time PCR System for fluorescence quantification; the amplified product was diluted to 500 ng/μL. The relative gene expression was calculated with the ^ΔΔ^Ct method. UBQ and AT1 were used as actins [36,37]. The primers used in this experiment are listed in Supplementary Table S1.

### 2.6. Statistical Analysis

Statistical analyses were performed using an ANOVA of Student’s *t*-test (*p* < 0.05; LSD and Duncan test) in GraphPad Prism8 software (version 8.0.2, GraphPad Software, San Diego, CA, USA). All experiments were repeated at least three times. *p =* 0.05 indicated significant values.

## 3. Results

### 3.1. zmcop1-1 and zmcop1-2 Are Two Loss-of-Function Mutants

**Figure 1:**
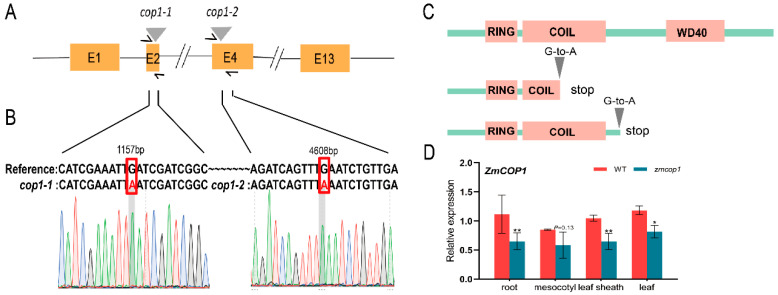
Genotyping expression patterns of *ZmCOP1*. (**A**) Illustration of the mutation sites in *zmcop1-1* and *zmcop1-2*. (**B**) Genotyping of *zmcop1-1* and *zmcop1-2*. B73 was used as a reference. As shown in the figure, G was mutated to A at the 1157th nucleotide in *zmcop1-1* and G was mutated to A at the 4608th nucleotide in *zmcop1-2*. (**C**) A model of the affected protein domains in *zmcop1-1* and *zmcop1-2*. (**D**) Expression patterns of *ZmCOP1* in different tissues (root, mesocotyl, leaf and leaf sheath). The maize seedlings were grown in darkness and at room temperature for 5 days. Data are means ± SD of at least three biological replicates; * *p* < 0.05, ** *p* < 0.01.

First, to understand the conservation of *COP1* in different species, we downloaded and analyzed *COP1* sequences. We found that *ZmCOP1* is highly similar to *AtCOP1* at the DNA sequence level (Figure S1). We then ordered two loss-of-function *zmcop1* mutants from the EMS mutant library (www.elabcaas.cn/memd/ (accessed on 14 September 2021)), naming them *zmcop1-1* and *zmcop1-2,* respectively. The two mutants were generated from B73 and self-fertilized for four generations. Through a Sanger sequencing analysis, we confirmed that both *zmcop1-1* and *zmcop1-2* were *zmcop1* stop-gain mutants that changed from TGA to TAA at the 1157th nucleotide and the 4608th nucleotide (Figure 1A,B), respectively. The mutation in *zmcop1-1* was located in the COIL helix domain; the base change led to the losses of the COIL and WD40 domains. The mutation in *zmcop1-2* was located between the COIL domain and the WD40 domain (Figure 1C), affecting the latter. It has been reported that WD40 is important in seedling and flower development as well as in light signal transmission and perception [38]. Thus, we propose that *zmcop1-1* and *zmcop1-2* may affect plant growth and development.

To understand the expression pattern of *ZmCOP1* in maize seedlings, we carried out an RT-qPCR analysis. We found that in 5-day-old etiolated seedlings in B73 and *zmcop1-1*, *ZmCOP1* was expressed in almost all tissues, indicating its role. The expression of *ZmCOP1* decreased significantly in *zmcop1-1* compared to in B73 by 42%, 40%, 38% and 31% in the root, mesocotyl, leaf sheath and leaf, respectively (Figure 1D).

### 3.2. zmcop1-1 and zmcop1-2 Shortened Mesocotyl Elongation

**Figure 2:**
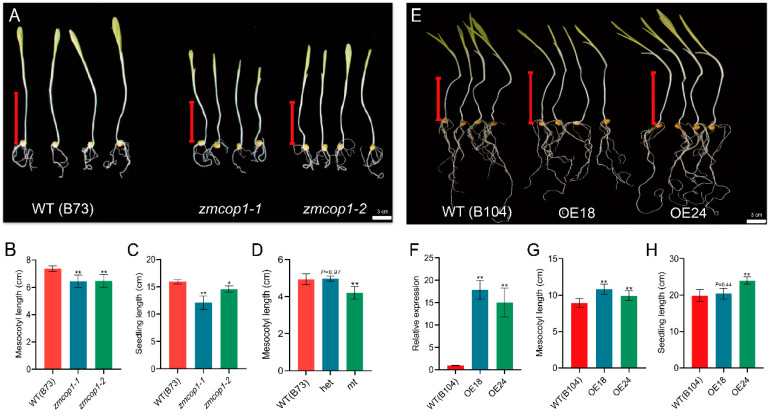
Phenotypic analyses of *zmcop1* mutants and overexpression transgenic lines. (**A**) Seedling phenotypes of wild-type B73, *zmcop1-1* and *zmcop1-2* grown in darkness for 7 days. Bars: 3 cm. (**B**) Quantification of mesocotyl length. (**C**) Quantification of seedling length. (**D**) Mesocotyl length segregation from a *zmcop1-1* heterozygous ear. (**E**) Seedling phenotypes of wild-type B104 and *ZmCOP1* overexpression lines (OE18, OE24, respectively) in darkness for 7 days. Bars: 3 cm. (**F**) Expressions of *ZmCOP1* in WT B104 (OE18, OE24), which was grown in darkness for 7 days. (**G**) Quantification of mesocotyl length. (**H**) Quantification of seedling length. Data are means ± SD of at least 10 biological replicates. Asterisks indicate significant differences in a two-way ANOVA (* *p* < 0.05, ** *p* < 0.01).

In order to study the function of *ZmCOP1* in mesocotyl elongation, we phenotypically analyzed the mesocotyl lengths of *zmcop1-1* and *zmcop1-2*, using B73 as a control (Figure 2A). When grown in the dark, B73 had a mesocotyl length of about 7.4 cm, while *zmcop1-1* and *zmcop1-2* each had a mesocotyl length of about 6.5 cm (Figure 2B). The mesocotyl length of *zmcop1-1* and *zmcop1-2* was significantly shorter than that of B73, with about a 12% reduction. The seedling length was 16.0 cm in the wild type, 12.1 cm in *zmcop1-1* and 14.6 cm in *zmcop1-2*. *zmcop1-1* and *zmcop1-2* showed significantly lower seedling lengths than the wild type (Figure 2C).

To understand whether the phenotype was caused by the *zmcop1* mutation, we performed a chi-square test using *zmcop1-1*. We found that in 121 seedlings, 100 showed the wild-type phenotype while 21 showed the short mesocotyl phenotype, which is inconsistent with the 3:1 segregation ratio (χ^2^ = 3.160, df = 1). These data indicate that the short mesocotyl phenotype is caused by one gene.

We then genotyped the *zmcop1-1* seedlings and found that the length was 4.9 cm for the wild-type genotype, 5.0 cm for the heterozygous genotype and 4.2 cm for the mutated homozygous genotype. A statistical analysis showed that there were no differences between the wild-type and the heterozygous genotype (Figure 2D), indicating that the mutation genotype is recessive. This result is consistent with the phenotype of *cop1* in *Arabidopsis thaliana* grown in the dark, which has a short hypocotyl length and plant height [9].

### 3.3. ZmCOP1 Overexpression Lines Showed Longer Mesocotyl Lengths

To understand whether overexpression of *ZmCOP1* promotes the length of etiolated seedling mesocotyls, we generated and planted transgenic lines while using B104 as a genetic background (Figure 2E). We gained more than 20 *ZmCOP1* OE lines and verified with RT-qPCR that the *ZmCOP1* in line 18 and line 24 was overexpressed more than tenfold compared with that in wild-type B104 (Figure 2F). We found that in darkness, the length of the etiolated seedling mesocotyl was about 10.8 cm in OE18, about 9.9 cm in OE24 and about 8.9 cm in wild-type B104 (Figure 2G). Thus, the mesocotyl lengths of the OEs were significantly longer than that of the wild type. We further measured the whole seedling length using these materials and found that the seedling length was 20.4 cm in OE18, 24.0 cm in OE24 and 19.8 cm in wild-type B104 (Figure 2H). The results showed that the high expression of *ZmCOP1* promoted the seedling hypocotyl length.

### 3.4. The Expressions of ZmHY5 and Other Light Genes Are Regulated by ZmCOP1

**Figure 3:**
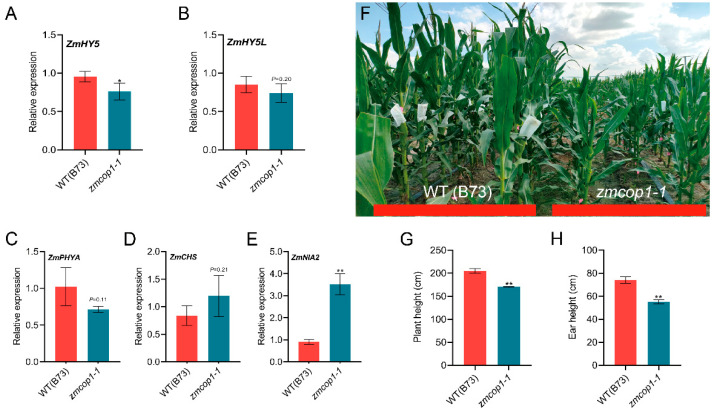
Gene expressions in and plant architecture of *zmcop1-1*. (**A**,**B**) Expressions of *ZmHY5* and *ZmHY5L* in wild-type B73 and *zmcop1* seedlings that were grown in darkness for 5 days. (**C**–**E**) Differential expressions of several light-regulating genes in wild-type B73 and *zmcop1* seedlings grown in darkness for 7 days. (**C**) Expressions of *ZmPHYA* in wild-type B73 and the *zmcop1-1* mutant. (**D**) Expressions of *ZmCHS* in wild-type B73 and the *zmcop1-1* mutant. (**E**) Expressions of *ZmNIA2* in wild-type B73 and the *zmcop1-1* mutant. (**F**) Plant architecture of *zmcop1-1* at silking time. (**G**) Quantification of plant height during the silking stage. (**H**) Quantification of ear height during the silking stage. Asterisks indicate significant differences between WT B73 and *zmcop1* using a Student’s *t*-test and a two-way ANOVA (* *p* < 0.05, ** *p* < 0.01).

HY5 and HY5L have been reported to interact with COP1 to inhibit the morphogenesis of the hypocotyl elongation of *Arabidopsis* seedlings [9,22]. To understand whether *ZmHY5* is regulated by *ZmCOP1*, we detected the expressions of *ZmHY5* and *ZmHY5L* in the 5-day-old etiolated seedlings. We found that the *ZmHY5* and *ZmHY5L* in *zmcop1-1* decreased by 20% and 13%, respectively (Figure 3A,B), indicating that the *ZmCOP1* led to expression changes for *ZmHY5* and *ZmHY5L* in the etiolated maize seedlings. However, contrasting the fact that *AtHY5* is degraded by *AtCOP1* [39], it seems that *ZmHY5* is stabilized by *ZmCOP1*. This needs to be studied further.

In order to understand whether *ZmCOP1* is involved in the absorption and utilization of light in maize, we identified the the expressions of several key light-regulating genes, such as *ZmPHYA*, *ZmCHS* and *ZmNIA2* [15]. We found that the expression of *ZmPHYA* in *zmcop1* was slightly but not statistically significantly lower than that in the WT (Figure 3C). The expressions of *ZmCHS* and *ZmNIA2* in *zmcop1* were higher than those in the WT, increased by 30% and 74%, respectively (Figure 3D,E). The phenomenon of *ZmCOP1* affecting the expression of photoregulatory factors indicated that the function of *ZmCOP1* is conserved for *AtCOP1*.

### 3.5. ZmCOP1 Affects Plant Height

In order to explore whether *ZmCOP1* affects plant development in the light, we carried out plant height phenotyping between WT B73 and *zmcop1* at the silking stage (Figure 3F). We found that the plant height was 204.8 cm in wild-type B73 and 170.4 cm in *zmcop1-1*. The plant height was lower by 17% in *zmcop1-1* than in the WT (Figure 3G). The ear height in the *zmcop1-1* mutant was also lower than that in the WT, reduced by 27% (Figure 3H). However, there was no significant difference in either the plant height or the ear height between the *zmcop1-2* mutant and wild-type B73; this needs to be studied further.

Chlorophyll is one of the most important photosynthetic pigments in plants, and its content directly affects the intensity of plant photosynthesis [40]. In order to clarify the effect of *ZmCOP1* on light absorption during maize’s growth period, we determined the relative content of chlorophyll in its leaves. We found that there was little difference in the SPAD values of wild-type B73, *zmcop1-1* and *zmcop1-2* in the first and ear leaves (Figure S2).

### 3.6. GO Analysis Showed That the DEGs Are Related to Hormone Signal Transduction

**Figure 4:**
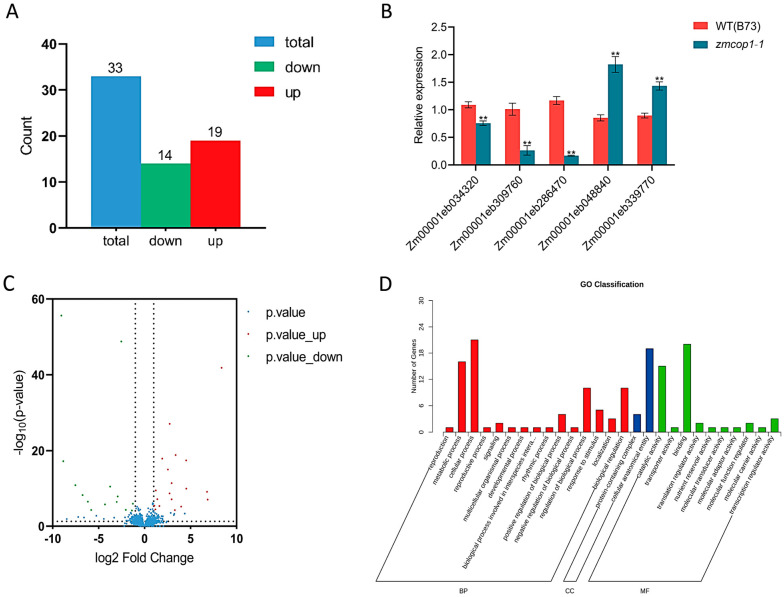
RNA-seq results showed that *zmcop1-1* mutation led to expression changes in some genes. (**A**) Differentially expressed genes between wild-type B73 and the *zmcop1-1* mutant. (**B**) Verification of the DEGs with RT-qPCR. Asterisks indicate significant differences between WT B73 and *zmcop1* using a Student’s *t*-test and a two-way ANOVA (** *p* < 0.01). (**C**) DEG volcano map. Red dots represent up-regulated differential genes in *zmcop1-1*, green dots represent down-regulated differential genes and blue dots represent non-differentially expressed genes. (**D**) GO bar chart. The abscissa denotes secondary GO entries, and the ordinate indicates the number of differential genes in the GO entries.

To further explore the functional mechanism of *ZmCOP1*, we performed a RNA sequencing analysis using *zmcop1-1* and wild-type B73. We found 33 DEGs, of which 19 were up-regulated and 14 were down-regulated (Figure 4A). To verify the expression patterns of the DEGs, we performed RT-qPCR. We confirmed that the results thereof were consistent with those of the RNA-seq (Figure 4B). A volcano map was used to show the overall distributions of differential genes in the WT and *zmcop1* (Figure 4C). Based on the expression patterns between the WT and *zmcop1*, we clustered the differentially expressed genes into groups (Figure S3). The genes that showed consistent expression patterns in all of the *zmcop1* replicates were speculated to have similar functions.

To understand the biological information of the DEGs, a GO (gene ontology) analysis was performed. According to the threshold of *p* ≤ 0.05, the DEGs were divided into three main functional categories: BP (biological process), CC (cellular component) and MF (molecular function) (Figure 4D). The regulations of biological (10 DEGs), metabolic (16 DEGs) and cellular processes (21 DEGs) were enriched in the BP category. The cellular anatomical entity (19 DEGs) was enriched in the CC category. The catalytic activity (15 DEGs) and binding (20 DEGs) were enriched in the MF category. The GO analysis showed that the DEGs are related to plant hormone signal transduction and metabolite biosynthesis.

### 3.7. KEGG Showed That DEGs Are Related to Hormone Signal Transduction

**Figure 5:**
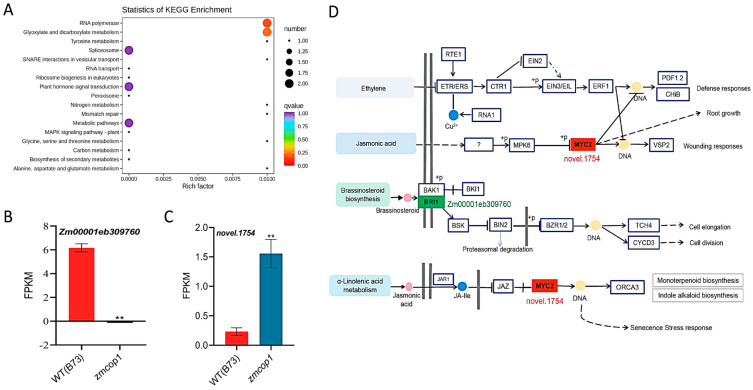
KEGG analysis of DEGs. (**A**) KEGG enrichment analysis scatter plot. The ordinate represents the KEGG path. The abscissa represents the rich factor. The larger the rich factor, the greater the degree of enrichment. The larger the point, the larger the number of differential genes enriched in the pathway. The redder the color of the dot, the more significant the enrichment. (**B**) Expression of *Zm00001eb309760* in RNA-seq. Asterisks indicate significant differences between wild-type B73 and the mutant material using a Student’s *t*-test (** *p* < 0.01). (**C**) Expression of *novle.1754* in RNA-seq. Asterisks indicate significant differences between wild-type B73 and the mutant material using a Student’s *t*-test (** *p* < 0.01). (**D**) KEGG pathway maps. The substances marked in red boxes represent up-regulated genes, while those marked in green boxes represent down-regulated genes.

To understand which biological processes DEGs participate in, KEGG (Kyoto Encyclopedia of Genes and Genomes) enrichment analyses were conducted (Figure 5A) [35]. We found that *ZmCOP1* regulates a wide range of KEGG pathways, such as those for MAPK hormone signal transduction, ribosome formation, RNA transport, glyoxylic acid and dicarboxylic acid metabolism and biosynthesis of secondary metabolites. These pathways are closely related to plant hormone production, genetic material changes and metabolite biosynthesis.

It has been reported that *ZmCOP1* regulates plant cell elongation and division by regulating the BR signal transduction pathway [41]. In *Arabidopsis thaliana*, both JA and BR play an important role in regulating cell and hypocotyl elongation [41,42,43]. We found that *Zm00001eb309760* was rarely expressed in *zmcop1* (Figure 5B). *Zm00001eb309760* is annotated to encode a leucine-rich repeat receptor-like protein kinase family protein. *Zm00001eb309760* may participate in the biosynthesis of BR. In addition, we found that a new gene, named *novel.1754*, was increased in the *zmcop1* mutant (Figure 5C). The KEGG analysis showed that *novel.1754* participates in the biosynthesis of jasmonic acid by regulating MYC2 and also regulates the α-linolenic acid metabolic pathway (Figure 5D). In summary, based on our KEGG results, we propose that *ZmCOP1* participates in the ETH, BR and JA plant hormone pathways.

## 4. Discussion

A previous study showed that *ZmCOP1* could restore the phenotype of an *atcop1-4* mutant in *Arabidopsis thaliana*, including the gene expressions for hypocotyl length, cotyledon openings, chlorophyll levels and light response [9]. In our study, the mesocotyl of the *zmcop1* mutants was significantly shorter than that of the WT in maize seedlings grown in the dark for 7 days (Figure 2B). By measuring the height of the seedlings, we found that the seedling height of the *zmcop1* mutants was also shorter (Figure 2C). However, unlike with the *atcop1* phenotype in *Arabidopsis thaliana*, we did not observe significant changes in the cotyledons at the maize seedling stage. We speculate that the function of *COP1* in *Arabidopsis thaliana* and maize is conservative, but there are also some differences.

COP1-HY5 forms the core complex that controls plant photomorphogenesis [22,44]. COP1 and HY5 play antagonistic roles in response to light signals and in the regulation of seedling morphogenesis. HY5 is a key transcription factor involved in the inhibition of hypocotyl elongation. We found that the contents of *ZmHY5* and *ZmHY5L* were both decreased in the *zmcop1* mutants (Figure 3A,B). It has been reported that *AtHY5* is degraded by *AtCOP1* [39], and our results showed that *ZmHY5* is stabilized by *ZmCOP1* at the RNA level. Whether ZmHY5 is degraded by ZmCOP1 at the protein level needs to be studied further.

Low expression of *AtCOP1* has been found to lead to differential expression of normal light-regulated genes in dark-treated materials. *atcop1* mutation inhibits photomorphogenesis and the elongation of hypocotyl [45,46]. In our research, through RT-qPCR, we confirmed that the expression of the light-regulated gene *ZmPHYA* in the *zmcop1* mutant seedlings treated in darkness for 7 days was slightly lower than that in the WT (Figure 3C). We speculate that some light-regulating genes in maize *zmcop1* are also affected, and that mutation of *zmcop1* affects plant morphogenesis.

It has been reported that *nia1* and *nia2* blossom and mature earlier than the WT in *Arabidopsis thaliana*, indicating that *AtNIA1* and *AtNIA1* are involved in the regulation of plant flowering [47]. We observed that the tasseling and flowering times of the *zmcop1* mutants were 3−5 days later than those of B73. Additionally, the expression of *ZmNIA2* was increased in the *zmcop1* mutants compared to in B73 (Figure 3E). Therefore, we speculated that the late flowering phenotype was caused by the high expression of *ZmNIA2* in the *zmcop1* mutants.

Studies have shown that under low light intensity, HY5 in Arabidopsis is down-regulated by COP1-mediated ubiquitination and degradation. BIN2 is partially inactivated with repression of the transcriptional activity of HY5. In contrast, BZR1 is accumulated (Li et al., 2020). As a result, the hypocotyl length is promoted by the enhanced transcriptional activity of the related genes. With an increase in light intensity, the elongation of Arabidopsis hypocotyls would be inhibited. Our RNA-seq results showed down-regulation of BRI1 in *zmcop1* mutants (Figure 5B). Considering the function of *AtCOP1*, we propose that *zmcop1* mutation first affects the downstream BR synthesis pathway, inhibiting cell elongation and division, and affects plant height afterward (Figure 5D).

## 5. Conclusions

We experimentally demonstrated the roles of *ZmCOP1* in maize morphogenesis. We found that *ZmCOP1* affects the elongation of maize mesocotyl in darkness and affects plant height in the light. We confirmed that *ZmCOP1* regulates the expressions of several key light factors in the same way as *AtCOP1*. Finally, we identified some DEGs and showed that *ZmCOP1* may control maize morphogenesis by regulating genes involved in the plant phytohormone pathway. Our data can be applied in maize performance improvement.

## Data Availability

Data will be made available on request.

## Supplementary Materials

The following supporting information can be downloaded at: https://www.mdpi.com/article/10.3390/life13071522/s1, Figure S1: Phylogenetic analysis of ZmCOP1. (A) Homology analysis of COP1 proteins in maize, sorghum, rice, grape, Arabidopsis, soybean and poplar. (B) Phylogenetic tree analysis showed that ZmCOP1 and AtCOP1 proteins are highly similar; Figure S2: Measurement of relative content of chlorophyll. (A) The leaf site of relative content of chlorophyll. (B) SPAD of the ear leaves. (C) SPAD of the first leaves. Data are mean ± SD of at least 20 plants.; Figure S3: RNA-seq results showed that zmcop1 mutation led to changes in the expression of some genes. (A) Differential gene clustering heat map. Abscissa denotes sample name and hierarchical clustering result, ordinate indicates differential gene and hierarchical clustering result. Red indicates high expression and green indicates low expression. (B) Kmeans cluster diagram. The Abscissa represents the sample, and the ordinate represents the standardized expression.; Table S1: The primers used in this experiment.
