# Supplementary material for: ZmCOP1 Regulates Maize Mesocotyl Length and Plant Height through the Phytohormone Pathways
Source: Life (Basel). 2023 Jul 7;13(7):1522. doi: 10.3390/life13071522 (PMC10381158; doi:10.3390/life13071522)
Supplement: Supplementary file 1 [file life-13-01522-s001.zip › Supplementary+Figure+S1.pdf]

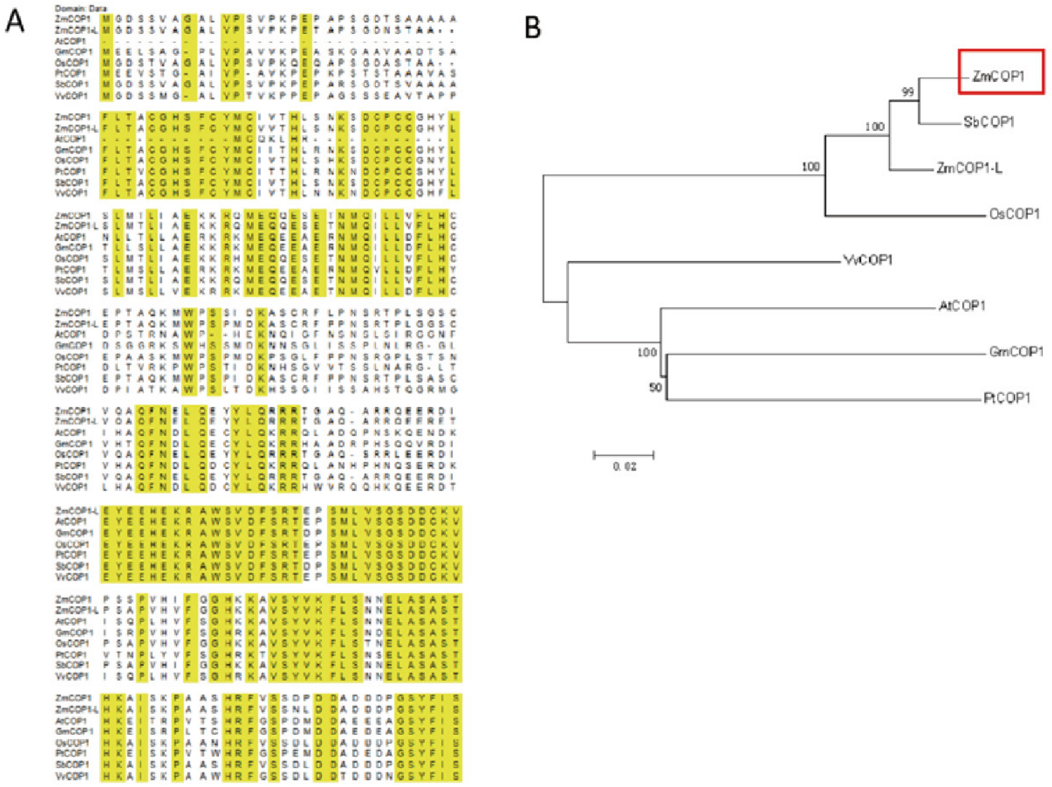

Fig. S1. Phylogenetic analysis of *ZmCOP1*.

(**A**) Homology analysis of COP1 proteins in maize, sorghum, rice, grape, Arabidopsis, soybean and poplar. (**B**) Phylogenetic tree analysis showed that ZmCOP1 and AtCOP1 proteins are highly similar.

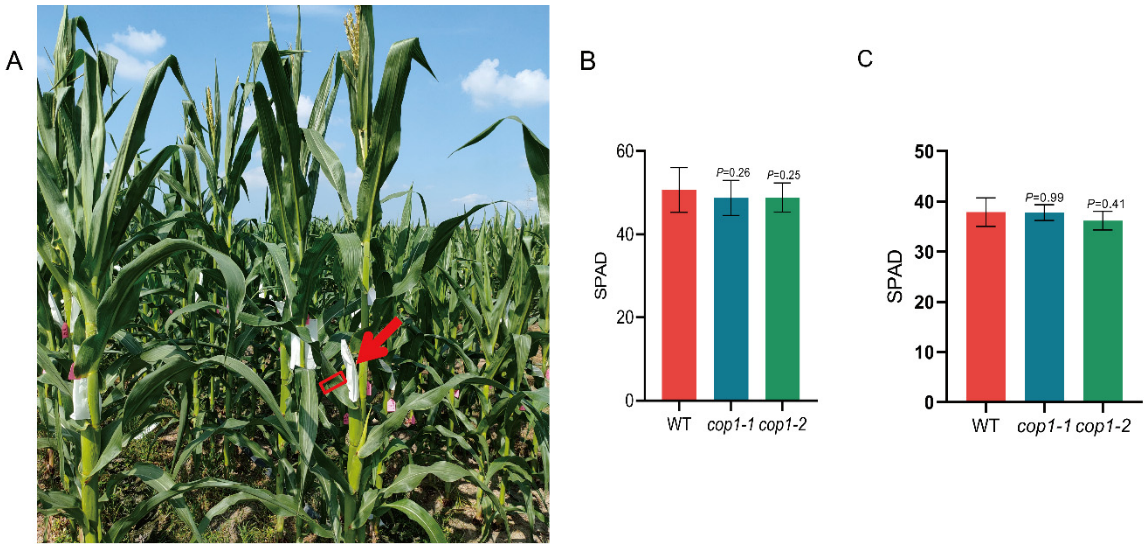

Fig. S2. Measurement of relative content of chlorophyll.

(A) The leaf site of relative content of chlorophyll. (**B**) SPAD of the ear leaves. (**C**) SPAD of the first leaves. Data are mean ± SD of at least 20 plants.

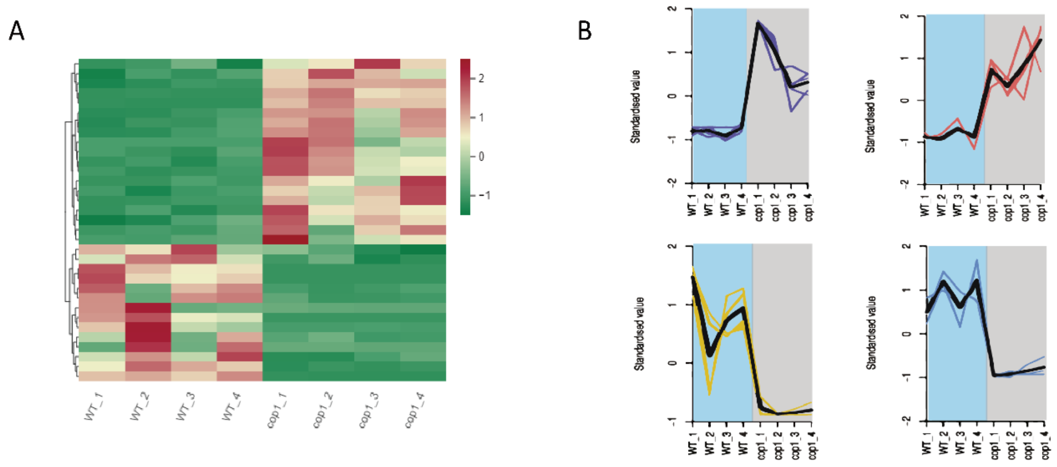

Fig. S3. RNA-seq results showed that *zmcop1* mutation led to changes in the expression of some genes.

(**A**) Differential gene clustering heat map. Abscissa denotes sample name and hierarchical clustering result, ordinate indicates differential gene and hierarchical clustering result. Red indicates high expression and green indicates low expression. (**B**) Kmeans cluster diagram. The Abscissa represents the sample, and the ordinate represents the standardized expression.
